# Comparison of the Effect of Alemtuzumab versus Standard Immune Induction on Early Kidney Allograft Function in Shiraz Transplant Center

**Published:** 2015-11-01

**Authors:** A. Khalafi-Nezhad, M. M. Sagheb, F. Amirmoezi, Z. Jowkar, A. R. Dehghanian

**Affiliations:** 1 Department of Hematology, Oncology and Stem Cell Transplantation, Shiraz University of Medical Sciences, Shiraz, Iran; 2 Shiraz Nephro-Urology Research Center, Shiraz University of Medical Sciences, Shiraz, Iran; 3 Student Research Committee, Cell and Molecular Research Group, Shiraz University of Medical Sciences, Shiraz, Iran; 4 Department of Operative Dentistry, School of Dentistry, Shiraz University of Medical Sciences, Shiraz, Iran; 5 *Department of Pathology Medicine, Shiraz University of Medical Sciences, Shiraz, Iran*

**Keywords:** Kidney transplantation, Immunosuppressive agents, Graft rejection, Alemtuzumab, Induction therapy

## Abstract

**Background:**

Induction therapy regimens classified as conventional immunosuppressive agents and lower doses of conventional agents combined with antibodies against T-cell antigens have been purposed to prevent acute rejection after renal transplantation. Various induction agents with different doses and durations have been suggested based on the risk profile of patients.

**Objective:**

To assess the acute rejection rate (total rate and based on the type of induction therapy regimen) during the first year after kidney transplantation, the type of acute rejection based on Banff classification and to determine the associations between rate of acute rejection, type of the rejection and induction therapy regimen.

**Methods:**

249 kidney transplant candidates were divided into two groups—low-risk patients (n=208) who received conventional immunosuppressive agents, and high-risk patients (n=41) who received alemtuzumab—and followed for one year to detect acute rejection first diagnosed clinically, and confirmed by percutaneous kidney biopsy based on Banff criteria.

**Results:**

The total incidence of acute rejection was 19.6% (20.7% of the low-risk and 14.4% of the high-risk patients). The most prevalent types of the acute rejection in patients treated with conventional immunosuppressive agents and patients received alemtuzumab as induction therapy were grade IB and grade IA, respectively. The incidence of acute rejection among recipients received a kidney from a deceased donor was 20.6% and grade IA was the most prevalent type (6.9%) whereas the most prevalent grade of acute rejection in patients who received living donor grafts was IB (8.3%).

**Conclusion:**

Despite the expected greater risk for acute rejection among high-risk patients, no significant difference was observed between low- and high-risk patients, which may be justified by the greater efficacy of alemtuzumab compared with standard triple induction therapy in reducing the rate of acute rejection.

## INTRODUCTION

One of the most common complications of renal transplantation is acute rejection, defined as rejection during the first year post-transplantation [1]. Induction therapy with potent immunosuppressive agents is ordered in the early stage of organ transplantation to reduce the risk of acute rejection [2]. Besides preventing acute renal transplant rejection, induction therapy, beginning intra-operatively or immediately post-operatively, has shown to reduce the overall dose of maintenance immunosuppressive regimens [2, 3]. Generally, induction therapies are classified as conventional immunosuppressive agents (usually cyclosporine, mycophenolate and methyl prednisolone) and as lower doses of conventional agents combined with antibodies directed against T-cell antigens such as rabbit anti-thymocyte globulin (thymoglobulin, Genzyme), a lymphocyte-depleting polyclonal antibody, basiliximab (Simulect, Novartis Pharmaceuticals), a non-lymphocyte-depleting monoclonal antibody targeting the interleukin-2 (IL-2) receptor, and more recently, alemtuzumab (Campath-1H, Berlex Laboratories), an anti-CD52 T-cell and B-cell monoclonal antibody [4-10].

It has been reported that several factors such as the type (live or deceased donor kidneys) and quality of donor kidneys (age of donor and donor kidney disease) may affect the renal allograft outcomes [11, 12]. According to the guidelines for kidney recipient care, risk factors for acute rejection are: 1) The number of human leukocyte antigen (HLA) mismatches (universal agreement); 2) older donor age (majority agreement); 3) younger recipient age (majority agreement); 4) panel reactive antibody (PRA) *>*10% (majority agreement); 5) African-American ethnicity (in the USA) (majority agreement); 6) blood group incompatibility (majority agreement); 7) delayed onset of graft function (majority agreement); 8) presence of a donor-specific antibody (majority agreement); and 9) cold ischemic time *>*24 hours (single study) [13].

There is no general consensus on the induction agent of choice, its dose or its duration [2]. According to a study by Chouhan, in order to reduce the incidence of acute rejection and the possible resultant graft loss from rejection, antibody induction therapy in adult patients with immunologic risk can be used. On the other hand, intravenous steroids (conventional therapy) without usage of any antibody are sufficient for induction therapy in low risk patients [14]. Oliaei found that adding thymoglobulin to the conventional immunosuppressant regimen in kidney transplantation decreased the occurrence of post-transplantation problems (signs of rejection, rise of creatinine, graft loses and delayed graft function) [4]. Jorge noted a significant reduction in the acute rejection rate with immunosuppressive therapy with basiliximab compared with triple immunosuppressive therapy (conventional) despite the same rate of graft or patient survival, death due to sepsis or incidence of post-transplantation malignancies [15]. In addition, in a study by Hanaway, alemtuzumab and rabbit antithymocyte globulin showed similar efficacy among high-risk patients. However, in low-risk patients induction therapy with alemtuzumab yielded a significant reduction in the acute rejection rate [16]. The same results of induction therapy were found with basiliximab and daclizumab by Naderi, *et al*. They also concluded that despite the possible reduction in acute rejection rate associated with induction therapy with monoclonal antibodies, they did not affect graft and patient survival rates compared with conventional therapies [17]. Nevertheless, Heldal showed that induction therapy treatment with IL-2 receptor antagonist had lower incidence of acute rejection and improved two-year graft survival in patients transplanted with kidneys from older deceased donors [18].

Although excellent results have been shown with alemtuzumab in some studies [16, 19], others have shown increased rates of acute rejection , decreased rates of graft survival after censoring of data on deaths, and increased rates of antibody-mediated rejection [20-22].

Considering the importance of induction therapy for the success of kidney transplantation, it will be useful to determine the efficacy of the different induction therapy regimens in the prevention of acute rejection and the effect of the regimen type on the severity of the rejection.

The objectives of this single-center, retrospective study, therefore, were to assess the acute rejection rate (total rate and based on the type of induction therapy regimen considered as alemtuzumab for high-risk patients and conventional induction therapy regimen for low-risk patients ) during the first year after kidney transplantation, the type of acute rejection based on Banff classification and to determine the associations between rate of acute rejection, pathologic type of the rejection and induction therapy regimen.

## MATERIALS AND METHODS

In a longitudinal, retrospective study a total of 249 patients (158 males and 91 females) who referred to Shiraz Transplant Center, Shiraz, Iran, for kidney transplantation and who received renal transplant from deceased or live donors between January 2011 and December 2012 was studied. Patients had a mean±SD aged of 38.6±13.74 (range: 18–69) years. An informed consent, approved by the Ethics Committee, Shiraz University of Medical Science, was obtained from all participants who granted permission for the use of their clinical data in the research. Participants were divided into two groups: low-risk (n=208) and high-risk (n=41). High-risk patients (for transplant rejection) were those with black race, a repeat transplant, and high panel-reactive antibodies (≥20%). The low-risk patients received conventional immunosuppressive agents (cyclosporine, 6 mg/kg; mycophenolate, 2 g; and methyl prednisolone, pulses of 500 mg iv, daily for 3 days and afterwards 1 mg/kg po); the high-risk patients received alemtuzumab (Campath-1H), 30 mg iv, at the time of transplantation). Maintenance immunosuppression consisting of standard triple therapy (prednisone, mycophenolate mofetil, tacrolimus) did not differ between the two groups. Each patient was followed for one year for acute rejection. Monitoring of the patients was as follows: daily while hospitalized, once a week for 3 months, twice a month for 6 months, and once a month up to 12 months post-transplantation. Acute rejection was first diagnosed clinically, defined as a creatinine rise of more than 20% of the baseline accompanied by fever (˃38 °C), pain over an enlarged kidney graft, increased kidney graft size, vascular resistance index (˃0.8) shown by color Doppler, and decreased urinary output, and then confirmed by percutaneous kidney biopsy based on Banff criteria [23].

Statistical analysis of the data was performed by SPSS^®^ for Windows^®^ ver18.0 (SPSS Inc, Chicago, IL, USA). χ^2^ test or Fisher’s exact test, when appropriate, and *Student’s t test* for unpaired data, were used to detect categorical variable differences and group differences, respectively. A p value <0.05 was considered statistically significant.

## RESULTS

Studied participants included 249 patients (158 [63.4%] males and 91 [36.6%] females) with a mean±SD age of 38.6±13.7 (range 18–69) years (Table 1). Based on the guidelines for kidney recipient care, 208 (83.5%) patients were considered low-risk. They were treated with conventional immunosuppressive agents; 41 (16.5%) patients were considered high-risk and received alemtuzumab.

**Table 1:** Demographic and transplant-related data

Mean±SD Age (Range, yrs)	38.6±13.7 (18–69)
Sex (M/F)	158/91
Risk of transplantation	
Low	208 (83.5%)
High	41 (16.5% )
Source of the donor	
Deceased	189 (75.9%)
Living	60 (24.1%)

The number of patients who received kidney transplants from deceased donors and living donor grafts were 189 and 60, respectively. The total incidence of acute rejection was 19.6% (20.7% in low-risk and 14.4% in high-risk patients). The maximum incidence of acute rejection based on Banff criteria [21] presented in Table 2, was related to grades IA (5.6%) and IB (5.6%); the lowest incidence of acute rejection was related to grade III (0.6%). The most prevalent types of the acute rejection in patients treated with conventional immunosuppressive agents and patients received alemtuzumab as induction therapy, were grade IB (n=12, 27.9%) and grade IA (n=3, 50%), respectively. Although patients received alemtuzumab, with the most prevalent grade as IA, had lower incidence of acute rejection compared to patients treated with conventional immunosuppressive agents, with the most prevalent grade as IB, no significant association was observed between different induction therapy regimens and the incidence of acute rejection or pathologic grade of the acute rejection. Of those patients who received kidney transplants from deceased donors, 151 (79.9%) were treated with conventional immunosuppressive agents and the remaining 38 patients received alemtuzumab as the induction therapy. Of living donor recipients, 57 (95%) were treated with conventional immunosuppressive agents and the remaining three patients (5%) received alemtuzumab. The incidence of acute rejection in recipients who received a kidney from a deceased donor was 20.6% (n=39) and grade IA was the most prevalent type (n=13, 6.9%), whereas the most prevalent grade of acute rejection in patients received living donor grafts was IB (n=5, 8.3%). Although the most prevalent grade of acute rejection was different among living and deceased donor recipients, there was no significant association between the type of renal transplant pathology and source of the donor. Also, no significant association was found between the incidence of acute rejection and source of the donor.

**Table 2:** Acute rejection rate in different group

Source of donor	Type of induction	Banff classification n (%)					
		IA	IIA	IB	IIB	III	No rejection
Living	Conventional induction	1 (2)	3 (5)	5 (9)	0 (0)	1 (2)	47 (82.5)
	Alemtuzumab	0 (0)	0 (0)	0 (0)	0 (0)	0 (0)	3 (100)
Deceased	Conventional induction	10 (6.6)	6 (4)	7 (4.6)	10 (6.6)	0 (0)	118 (78.1)
	Alemtuzumab	3 (8)	1 (3)	2 (5)	0 (0)	0 (0)	32 (84)
Total		14 (5.6)	10 (4)	14 (5.6)	10 (4)	1 (0.4)	200 (80.4)

## DISCUSSION

With an incidence of 20%–50%, acute rejection is one of the most common complications of renal transplantation [24]. Besides increasing the incidence of early kidney non-function, it is considered an important risk factor for late kidney graft loss eventually leading to the increased treatment cost and declined half-life of the transplant by four years compared with patients without any acute rejection. Therefore, any attempt to prevent and decrease early stage acute rejection would be valuable to increase the long-term survival of patients and grafts [25]. To date, various inductors have been adopted to decrease the incidence of acute rejection rate. Since 1998, alemtuzumab, a humanized anti-CD52 depleting monoclonal antibody leading to profound depletion of B and T lymphocytes, monocytes, NK cells, macrophages, and dendritic cells, has been administrated successfully as an induction therapy agent for organ transplantation [26]. CD52 glycoprotein is not expressed on platelets, granulocytes, erythrocytes, and hematopoietic stem cells. Complement-mediated cytolysis, apoptosis, and antibody-mediated cytotoxicity are the mechanisms of action of Campath-1H [27].

At our institution, standard induction therapy regimen for kidney transplantation is conventional immunosuppressive therapy for low-risk patients whereas alemtuzumab is administered to high-risk patients.

In our study the acute rejection rate in high-risk patients (14.4%) was lower than that in low-risk patients (20.7%), although the difference was not statistically significant. Despite not being statistically different, the severity of the acute rejection in high-risk patients (IA) was lower than that of low-risk patients (IB). Also, there was no statistically significant correlation between the incidence of acute rejection, the type of renal transplant pathology and source of the donor.

The various maintenance therapy regimens among different studies make direct comparison between alemtuzumab and other induction therapy regimens somehow difficult.

Excellent short-term survival, a low incidence of associated infectious complications, and lower rates of acute rejection have been reported for alemtuzumab as an induction therapy agent [28, 29]. Other advantages of alemtuzumab include a single-dose application, good early acute rejection prophylaxis, and less need for calcineurin inhibitors [30]. Furthermore, the ability to maintain patients on steroid-free regimens has been associated with alemtuzumab [31]. Vathsala, *et al*, showed results similar to our study where induction therapy with alemtuzumab and cyclosporine was as effective as standard therapy, which consisted of cyclosporine, azathioprine, and steroid therapy. In contrast to our study, a second dose of alemtuzumab was administered 24 hours post-operatively [32]. 

Moreover, Lü, *et al*, compared the efficacy and safety of alemtuzumab, as immune induction therapy, with anti-thymocyte globulin in highly sensitized kidney transplant recipients in a randomized clinical trial and found alemtuzumab as an effective and safe induction agent with an acceptable acute rejection rate (18.2%) [19].

Our protocol was different from the previously mentioned articles; low-risk patients were only treated with conventional immunosuppressive agents (prednisone, mycophenolate mofetil, and tacrolimus) and high-risk patients were induced by alemtuzumab. The total rate of acute rejection was 19.6% in our study, which was comparable to a previous study by Baez, *et al*, that showed a 17% incidence of acute rejection at one year. Moreover, they concluded that alemtuzumab was safe and effective for steroid-free maintenance immunosuppression in renal transplantation. In addition, the severity of acute rejection in their study was mild (IA or IB), which was similar to our findings [30]. Steroid-free immunosuppressive therapy leads to the reduced rate of adverse metabolic effects, such as post-transplant diabetes mellitus, osteoporosis, and surgical wound infections as well as decreased risk of developing infections due to cytomegalovirus with this protocol and excellent long-term outcomes [33, 34]. In another randomized clinical trial by Hanaway, *et al*, alemtuzumab showed less frequent biopsy-confirmed acute rejection than conventional therapy. Due to the similar efficacy of the alemtuzumab and rabbit antithymocyte globulin in high-risk patients, the apparent superiority of alemtuzumab was limited to low-risk patients for transplant rejection. An alternative induction immunosuppressive therapy regimen is administration of anti-IL-2 receptor antibodies, namely basiliximab. Hanaway, *et al*, also found greater severity of rejection and graft-rejection rate with basiliximab as compared with alemtuzumab induction [16].

There are some reports about later occurrence of acute rejection after alemtuzumab induction with a greater risk of hormonal rejection [35]. This issue can justify our results regarding higher incidence of acute rejection rates, although statistically insignificant, in low-risk patients compared to high-risk patients in one-year follow-up period, which may be different in longer follow-up due to the possible later occurrence of acute rejection after alemtuzumab induction. 

Considering the cost of alemtuzumab, one of the limitations of our retrospective study was administration of alemtuzumab only to high-risk patients. However, for more accurate comparison between alemtuzumab and conventional induction therapy regimen, more prospective studies with alemtuzumab in the same groups are recommended. More longitudinal studies on larger populations and with longer follow-up are necessary to investigate possible efficacy and side-effects of alemtuzumab in both high- and low-risk patients compared with other induction therapy regimens such as basiliximab.

In conclusion, despite the expected greater risk for acute rejection among high-risk patients, no statistically significant difference was observed among low- and high-risk patients, which can be justified by the greater efficacy of alemtuzumab compared with standard triple induction therapy in reducing the rate of acute rejection.
